# Acupuncture for postoperative delirium (POD): A systematic review and meta-analysis protocol

**DOI:** 10.1097/MD.0000000000023822

**Published:** 2021-01-22

**Authors:** Chanwoo Joo, Seunghoon Lee, Jung Won Kang, Jae-Dong Lee

**Affiliations:** a Department of Clinical Korean Medicine, Graduate School, Kyung Hee University; b Department of Acupuncture & Moxibustion Medicine, Kyung Hee University Korean Medicine Hospital; c Department of Acupuncture and Moxibustion, College of Korean Medicine, Kyung Hee University, Seoul, Republic of Korea.

**Keywords:** acupuncture, meta-analysis, postoperative delirium, protocol, systematic review

## Abstract

**Background:**

Postoperative delirium (POD) is a form of delirium that is newly diagnosed after a surgical procedure. This study aims to examine the effectiveness and safety of acupuncture treatment for POD in patients who underwent surgery.

**Methods:**

Randomized controlled trials for patients diagnosed with POD using validated delirium assessment scales will be included in this review. Electronic databases, such as MEDLINE, EMBASE, CENTRAL, CINAHL (English DB), CNKI, Wanfang, VIP (Chinese database), KoreaMed, RISS, KISS, DBpia, OASIS (Korean DB), and J-STAGE (Japanese DB) will be searched without language limitation from their inception to October 2020. The intervention group will include patients who have received any type of acupuncture treatment for POD. The control group will include individuals with no treatment, sham acupuncture treatment, and conventional treatment. The primary outcome is the incidence of POD in each study. Quality assessment will be performed using the Cochrane risk of bias tool. A meta-analysis will be performed to pool the estimated effect.

**Conclusion:**

This study will provide evidence for acupuncture as a potential treatment for POD, in researchers, patients, and policy makers.

**Dissemination:**

The result of the study will be disseminated through posters, press releases, conference presentations, and peer-reviewed papers.

**Trial registration number:**

OSF 2020: (https://osf.io/usvdg)

## 1 Introduction

### 1.1 Description of the condition

Delirium is a state where the patient experiences acute disturbance of consciousness with decreased ability to focus or maintain attention, accompanied by cognition change and perceptual disturbances.^[1]^ Postoperative delirium (POD) is characterized in patients newly diagnosed with a delirium after surgical procedures and anesthesia.^[2]^ Although there is no definite mechanism for POD, some possible causes are known, such as inflammation, neurotransmitter, chronic stress, metabolic disorders, electrolyte disorders, and sleep-wake disturbance.^[3]^ In the US, the prevalence of delirium in the general medicine population is 10% to 24%, while a higher prevalence of 37% to 46% has been observed in the surgical population.^[2]^ Depending on the patient's age, type of surgery, and various medical settings, the prevalence of POD varies from 9% to 87%.^[4]^ Although it is often fatal to patients, POD is still under-recognized among health care providers. POD can lead to increased health care cost, make poor prognosis after surgery, and lengthened hospital stay.^[5]^

### 1.2 Description of the intervention

Acupuncture is a procedure that uses needles with various manipulations to stimulates specific points (e.g., acupuncture points, myofascial points, or tender points). Acupuncture has been used for treating internal disorders, musculoskeletal diseases, and other medical problems, since 3000 years ago in East Asian countries, such as Korea, Japan, and China.^[6]^

Among its various uses, acupuncture has been used mostly for the treatment of acute and chronic pain.^[7]^ There has been a lot of clinical evidence on pain management,^[8]^ especially on musculoskeletal pain, including low back pain^[9]^ and myofascial pain syndrome.^[10]^ Recently, the clinical application of acupuncture treatment for mental disorders, including insomnia, anxiety, depression, schizophrenia, dementia, cognitive impairment, and delirium, is gradually increasing.^[11]^

### 1.3 How the intervention might work

Acupuncture mechanisms have not been fully explained, and the best-known mechanism is the pain management, which is related to adenosine A1 receptor,^[12]^ gate control theory,^[13]^ and descending pathway.^[14]^ There are some ongoing studies^[15,16]^ on the effects of acupuncture on mental disorders such as neurocognitive disease. A review^[17]^ suggested that the mechanism of acupuncture for neurocognitive disorders could be related to the attenuation of systemic inflammation and neuroinflammation, improvement of synaptic plasticity, reduction of oxidative stress level, neuronal injury, anesthetic agent usage, and promotion of patient recovery.

### 1.4 Why it is important to perform this review

The pharmacological treatments for delirium are the typical and atypical antipsychotics.^[2]^ Although haloperidol is frequently used in delirium treatment, it has considerable side effects, including long QT syndrome and movement disorders.^[18]^ Known for significantly reducing the prevalence of POD, dexmedetomidine^[19]^ has also been reported to have several side effects on cardiovascular and parasympathetic side effects, such as bradycardia, hypotension, and dry mouth.^[20]^

Although nonpharmacological treatment has insufficient evidence,^[21]^ acupuncture has been reported as an alternative intervention for preventing POD.^[22]^ To produce sedation without complications, some studies have suggested that acupuncture could be a potential intervention for POD.^[23,24]^ However, there are no systematic reviews studying the effectiveness of acupuncture on this condition.

### 1.5 Objective

The objective of the present study is to determine the effectiveness of acupuncture treatment for POD in patients who underwent surgery in comparison with no treatment, sham, and conventional treatment.

## 2 Methods

### 2.1 Study registration

The protocol of the review was registered (https://osf.io/usvdg) in November 2020.

### 2.2 Inclusion criteria

#### 2.2.1 Types of studies

A prospective randomized controlled trial (RCT) of acupuncture dealing with POD will be included in this review. Qualitative studies, observational studies, and non-RCTs will be excluded. The language of studies will not be restricted.

#### 2.2.2 Types of participants

Patients older than 18 years with no history of previous delirium and neurocognitive problems before admission, but with newly diagnosed delirium after receiving any type of surgery (e.g., spine surgery, cardiac surgery, and replacement surgery) will be included. This study will include the occurrence of delirium within 7 days after the completion of anesthesia or surgery.^[25]^

#### 2.2.3 Types of interventions

Any type of acupuncture treatments [e.g., manual acupuncture, electroacupuncture, transcutaneous electrical acupoint stimulation (TEAS), transcutaneous electrical nerve stimulation (TENS), pharmaco-acupuncture, auricular acupuncture, acupressure, and dry needling] will be included. Other types of Korean medicine treatments, such as moxibustion, cupping therapy, and herbal medicine, will be excluded.

The control intervention will include no treatment, sham acupuncture, and conventional treatment. If the acupuncture group simultaneously received conventional treatment, which is used in the control and acupuncture treatment, the trial will be included in this review.

#### 2.2.4 Types of outcome measures

##### 2.2.4.1 Primary outcomes

(1) Incidence of delirium (frequency): from postoperative days 1 to 7, the occurrence of delirium will be counted. Delirium will be diagnosed using validated assessment methods of delirium (e.g., CAM, ICU-CAM, NEECHAM, or K-DRS).^[26]^

##### 2.2.4.2 Secondary outcomes

(1) Duration of delirium
(2) Intensity of delirium
(3) Severity of delirium
(4) Length of hospital stay
(5) Adverse effects

### 2.3 Search methods for identification of studies

#### 2.3.1 Electronic searches

Thirteen databases will be used in the present study: Ovid MEDLINE (1946 to October 2020), Cochrane Central Register of Controlled Trials (CENTRAL) (*The Cochrane Library*, 2020 Issue 10), EMBASE (1980 to October 2020), Cumulative Index to Nursing and Allied Health Literature (CINAHL, 1982 to October 2020), 3 Chinese databases [China National Knowledge Infrastructure (CNKI), Wanfang, and Chongqing VIP Chinese Scientific Journals Full-text Database], 5 Korean databases [KoreaMed, Research Information Service System (RISS), Korean Studies Information Service System (KISS), Database Periodical Information Academic (DBpia), and Oriental Medicine Advanced Searching Integrated System (OASIS)], and 1 Japanese database [Japan Science and Technology Information Aggregator Electronic (J-STAGE)].

#### 2.3.2 Search for other resources

For ongoing and recently completed studies, the World Health Organization International Clinical Trials Registry Platform will be searched. Reference lists in the relevant publications will be manually checked for additional eligible trials.

#### 2.3.3 Search strategy

The search strategy consists of 3 parts: postoperative (e.g., after surgery, postoperative, and post-surgery), delirium (e.g., delirium, acute confusion, and metabolic encephalopathy), and acupuncture (e.g., acupuncture, acupuncture therapy, electroacupuncture, TEAS, and TENS) (see Table, Supplemental Content, which illustrates the search strategy for Ovid MEDLINE).

### 2.4 Data collection and analysis

#### 2.4.1 Selection of studies

Two researchers (CJ and SL) will independently read the title and abstract of articles and screen eligible articles using predefined criteria on standard eligibility form. Disagreements about the selection of studies will be resolved through discussion, and a third researcher will resolve the disagreement that remains. All flow processes will be summarized in a Preferred Reporting Items for Systematic Reviews and Meta-Analysis (PRISMA)-compliant flow chart (http://www.prisma-statement.org).

#### 2.4.2 Data extraction and management

Using a standard data extraction form, 2 researchers (CJ and SL) will independently extract the data (e.g. sample size, author, year of publication, nation, study design, participants, acupuncture intervention, control intervention, outcome measures, main outcomes, and adverse events). Using the Revised Standards for Reporting Interventions in Clinical Trials of Acupuncture (STRICTA) guidelines, acupuncture treatment will be extracted. If they cannot reach a consensus on derived results, it will be resolved by discussion between researchers.

#### 2.4.3 Assessment of risk of bias

Researchers will individually perform quality assessment using the Cochrane risk of bias (ROB) tool on the Cochrane Handbook for Systematic Reviews of Interventions.^[27]^ The Cochrane ROB tool has 7 domains: random sequence generation, allocation concealment, blinding of participants, blinding of outcome assessors, incomplete outcome data, selective outcome reporting, and other sources of bias. The ROB will be categorized into 3 levels: low, high, and unclear ROB. Any disagreement will be resolved through discussion or consultation among the researchers.

#### 2.4.4 Measures of treatment effect

For dichotomous data, the relative ratio with 95% confidence intervals (95% CIs) will be used to measure the treatment effect. For continuous data, the mean difference with 95% CIs will be used to measure the treatment effect. The standardized mean difference will be used in continuous outcomes scales that do not correspond among studies.

#### 2.4.5 Unit of analysis issues

To prevent the carry-over effect, only the data from the first period of the cross-over study will be used. Cluster RCTs will not be included.

#### 2.4.6 Dealing with missing data

The original study authors will be contacted, and missing or additional data will be requested. If the missing data cannot be obtained, only the available data will be analyzed. In the discussion, the potential impact of missing data will be addressed.

#### 2.4.7 Assessment of heterogeneity

The between-trial heterogeneity will be used to assessed preferentially by visual inspection of the forest plot, and a χ^2^ test with a significance level of *P* < .10 will define the presence of heterogeneity.

In addition, the *I*^2^ statistic will be assessed to quantify the inconsistencies among the studies, where a value of more than 50% indicates a meaningful heterogeneity. *I*^2^ of 0% to 40% may be unimportant, 30% to 60% may have moderate heterogeneity, 50% to 90% may have substantial heterogeneity, and 75% to 100% may have considerable heterogeneity.

#### 2.4.8 Assessment of reporting biases

To examine reporting biases, a funnel plot will be used when more than 10 studies are available.

#### 2.4.9 Data synthesis

To calculate a summary estimate, the meta-analysis will be conducted using Review Manager software (RevMan, version 5.4 for Windows; the Nordic Cochrane Centre, Copenhagen, Denmark). The pooled effect estimate will be determined by calculating the weighted average. A fixed-effect model will be used as the main analysis. If there is a considerable heterogeneity that cannot be fully not explained by variation in methodological and clinical features, data synthesis will be performed carefully.

#### 2.4.10 Subgroup analysis and investigation of heterogeneity

When sufficient numbers of studies is available, subgroup analysis will be performed to identify the heterogeneity among studies according to the following:

(1) Type of acupuncture stimulation (e.g., penetration vs non-penetration and manual vs electrical)
(2) Type of control (e.g., no treatment, sham acupuncture, conventional medicine, and other additional treatments.)
(3) Timing of acupuncture treatment (e.g., preoperative, intraoperative, postoperative, and mixed operative)
(4) Characteristics of surgery (e.g., surgery type, duration of surgery, duration of anesthesia, and consumption of anesthesia)

#### 2.4.11 Sensitivity analysis

In order to determine whether the findings are robust, sensitivity analysis will be performed as follows:

(1) Methodological qualities (e.g., whether random sequence generation and allocation concealment are adequately conducted or not)
(2) Statistical methods (e.g., fixed-effects model vs random-effects model)

#### 2.4.12 Summary of evidence

The main outcomes (primary outcome and secondary outcome) will be presented in the “Summary of findings” tables. To evaluate the quality of evidence in the main outcomes, Grading of Recommendations Assessment, Development, and Evaluation (GRADE) approach will be used.

### 2.5 Ethics and dissemination

Because the current study is a systematic review, informed consent and ethical approval were not needed. The results of the present study will be distributed through posters, press releases, conference presentations, and peer-reviewed papers.

## 3 Discussion

This is the protocol of a systematic review that will investigate acupuncture as an effective and safe treatment for patients with POD.

Surgical procedures often cause several discomforts, such as pain, nausea, wound, ileus, and mental disorders.^[28]^ Although these postoperative complications are often preventable, they frequently lengthen hospital stay and increase health care cost.^[29]^ In this context, postoperative care to prevent postoperative complications is an important concern for health care providers. POD is a crucial postoperative complication that incurs a significant social cost (e.g., lengthening hospital stay and increasing health care cost) and patient's health problems (e.g., poor prognosis after surgery, increasing risk of dementia, increasing readmission rate, and increasing all-cause mortality).

Acupuncture has been used in several postoperative care settings. There are some reviews mentioning that acupuncture has therapeutic effects on postoperative complications, such as acute postsurgical pain,^[30]^ opioid consumption,^[31]^ nausea, vomiting,^[32]^ and ileus.^[33]^ Although the mechanism of acupuncture for POD is not well-understood, based on clinical practice and studies wherein acupuncture has been used for neurocognitive disorders,^[23,24]^ acupuncture might be a potent, safe, and effective nonpharmacological treatment for POD.

Therefore, this present study will provide the evidence for the effectiveness and safety of acupuncture for POD. These findings will present recommendation to clinicians, health care policy makers, and patients on the use of acupuncture for POD. Moreover, this study will enable researchers will design further clinical studies.

## Acknowledgments

The author would like to thank Professor Kun Hyung Kim for encouraging suggestions and helpful comments.

## Author contributions

**Conceptualization:** Seunghoon Lee, Chanwoo Joo.

**Funding acquisition:** Seunghoon Lee.

**Methodology and project administration:** Seunghoon Lee, Chanwoo Joo.

**Writing – original draft:** Seunghoon Lee, Chanwoo Joo.

**Writing – review & editing:** Seunghoon Lee, Jung won Kang, Jae-Dong Lee.
